# Introduction to the Special Issue on Taking Home Visiting to Scale: Findings from the Maternal, Infant, and Early Childhood Home Visiting Program State-Led Evaluations

**DOI:** 10.1007/s10995-018-2539-5

**Published:** 2018-06-19

**Authors:** Nicole Denmark, Kyle Peplinski, Mariel Sparr, Judy Labiner-Wolfe, Susan Zaid, Pooja Gupta, Kassie Mae Miller

**Affiliations:** 1 US Department of Health and Human Services, Administration for Children and Families, Office of Planning, Research, and Evaluation, 330 C St SW, Washington, DC 20201 USA; 2 US Department of Health and Human Services, Health Resources and Services Administration, Maternal and Child Health Bureau, 5600 Fisher Lane, Rockville, MD 20857 USA; 3 James Bell Associates, 3033 Wilson Boulevard, Suite 650, Arlington, VA 22201 USA

**Keywords:** Early childhood home visiting, Program evaluation, Implementation research, Evidence-based programming

## Abstract

The Maternal, Infant and Early Childhood Home Visiting (MIECHV) Program is a two-generation approach to supporting healthy families through home visits during pregnancy and early childhood. All states and territories receiving MIECHV funding are encouraged to evaluate their programs. This special issue highlights evaluations from 11 awardees—Arkansas, Florida, Illinois, Iowa, Maryland, Massachusetts, Michigan, New Jersey, Oregon, Pennsylvania, and Tennessee. With the wide expansion of home visiting since the onset of MIECHV, the state-led evaluations contribute to the understanding of replication and scale-up of evidence-based home visiting.

The Maternal, Infant and Early Childhood Home visiting (MIECHV) Program is a two-generation approach to supporting healthy families through home visits during pregnancy and early childhood. MIECHV is administered by the Health Resources and Services Administration in collaboration with the Administration for Children and Families. With a $2.7 billion federal investment since the program launched in 2010, MIECHV has provided over 3.3 million home visits to at-risk families in all 50 states, Washington DC, and five territories. In 2016, the program served approximately 160,000 parents and children in 893 counties across the United States.

Statutory authority for the program (42 U.S.C. § 711 2018) ensures both a grounding in, and continuous learning from, evaluation. Program requirements include that the majority of grant funding is used to implement home visiting models that have undergone rigorous evaluation and demonstrated their effectiveness on outcomes important to healthy families. Up to 25% of grant funds may be used to implement a promising, but not evidence-based, model and must be accompanied by a rigorous evaluation. All states and territories receiving MIECHV funding are encouraged to evaluate their programs. Additionally, 3% of MIECHV funding each year supports a continuous agenda of research and evaluation to expand understanding of the implementation and effectiveness of home visiting programs.

Since 2011, a total of 124 state-led evaluations have been conducted. Fifty-eight of these evaluations have been completed. States and the District of Columbia are utilizing a variety of evaluation designs to address questions relevant to their local program contexts and to the broader home visiting field, including implementation/fidelity designs; systems change evaluations; matched comparison designs; and randomized control trials. Local evaluations explore several topics, including: collaboration and coordination with other service sectors; workforce development; program enhancements, innovations, and promising approaches; program quality and fidelity; and family engagement. The local evaluations are funded through allocation of up to 10% of total grant budgets, and awardees work with external evaluators. Throughout the course of the evaluation, awardees receive technical assistance (TA) from the Design Options for Home Visiting Evaluation (DOHVE) project, federal evaluation staff, and federal project officers to design and implement rigorous evaluations.

The MIECHV state-led evaluations help answer questions awardees have about their programs while potentially adding to the larger evidence base on home visiting. Specifically, with the wide expansion of home visiting since the onset of MIECHV, the state-led evaluations can contribute to our understanding of replication and scale-up of evidence-based home visiting. Furthermore, by studying the implementation of evidence-based home visiting in new settings and with different cultural and local contexts, the evaluations add to the external validity of the current evidence base. Rigorous impact studies of promising approach models can add to the literature on what works, as do studies of add-ons to traditional evidence-based models. Finally, as the state-led evaluations are required to address questions of interest to programs, themes in their focus contribute to our understanding of implementation challenges and supports.

This special issue highlights evaluations from 11 awardees—Arkansas, Florida, Illinois, Iowa, Maryland, Massachusetts, Michigan, New Jersey, Oregon, Pennsylvania, and Tennessee. Initial abstracts were selected based on the rigor and potential relevance of the evaluations for the field. In addition to these criteria, guest editors chose abstracts representing a diversity of topics, methods, geographical distributions, and home visiting models seen in the larger set of MIECHV evaluations. The resulting articles cover four broad topics- understanding and enhancing program quality and fidelity (Iowa, Michigan, and Massachusetts), family engagement (Arkansas, New Jersey, and Oregon), workforce development (Florida and Maryland), and impacts of home visiting, including evidence-based models, promising approaches, and enhancements to models, on key maternal and child health outcomes (Pennsylvania, Tennessee, and Illinois).

State-led evaluations provide valuable information for the MIECHV program and the broader field of home visiting. Thus the Health Resources and Services Administration continues to encourage awardees to pursue questions of programmatic interest. The local evaluations provide an unprecedented body of findings around the implementation and effectiveness of home visiting, complementing ongoing federal and non-federal efforts to learn from and scale up evidence-based programs.

## References

[CR1] Social Security Act of 1935, as amended, 42 U.S.C. § 711(c)) (2018).

