# Correction to: Introduction to the Special Issue on Taking Home Visiting to Scale: Findings from the Maternal, Infant, and Early Childhood Home Visiting Program State-Led Evaluations

**DOI:** 10.1007/s10995-018-2616-9

**Published:** 2018-08-22

**Authors:** Nicole Denmark, Kyle Peplinski, Mariel Sparr, Judy Labiner-Wolfe, Susan Zaid, Pooja Gupta, Kassie Mae Miller

**Affiliations:** 1 US Department of Health and Human Services, Administration for Children and Families, Office of Planning, Research, and Evaluation, 330 C St SW, Washington, DC 20201 USA; 2 US Department of Health and Human Services, Health Resources and Services Administration, Maternal and Child Health Bureau, 5600 Fisher Lane, Rockville, MD 20857 USA; 3 James Bell Associates, 3033 Wilson Boulevard, Suite 650, Arlington, VA 22201 USA

## Correction to: Maternal and Child Health Journal 10.1007/s10995-018-2539-5

The article “Introduction to the Special Issue on Taking Home Visiting to Scale: Findings from the Maternal, Infant, and Early Childhood Home Visiting Program State-Led Evaluations”, written by Nicole Denmark, Kyle Peplinski, Mariel Sparr, Judy Labiner-Wolfe, Susan Zaid, Pooja Gupta and Kassie Mae Miller, was originally published electronically on the publisher’s internet portal (currently SpringerLink) on 19 June 2018 without open access. With the author(s)’ decision to opt for Open Choice the copyright of the article changed on 18 July 2018 to © The Author(s) 2018 and the article is forthwith distributed under the terms of the Creative Commons Attribution 4.0 International License (http://creativecommons.org/licenses/by/4.0/), which permits use, duplication, adaptation, distribution and reproduction in any medium or format, as long as you give appropriate credit to the original author(s) and the source, provide a link to the Creative Commons license and indicate if changes were made.

The original article has been corrected.

